# Evolution of Fused Silica Subsurface Damage in Chemical Etching and Its Influence on Surface Loss

**DOI:** 10.3390/s26082449

**Published:** 2026-04-16

**Authors:** Xiaoqiang Zhang, Chengkui Zu, Lidong Wang, Xuefu Song, Yuancheng Sun, Xiurong Du, Yongchang Zhu

**Affiliations:** 1 Glass-Based Functional Materials Technology Innovation Center, China Building Materials Academy, Beijing 100024, China; 2 School of Materials Science and Engineering, Harbin Institute of Technology, Harbin 150001, China

**Keywords:** fused silica resonator, subsurface damage, chemical etching, mechanical quality factor, surface loss

## Abstract

**Highlights:**

**What are the main findings?**

- Elucidated the mechanism of surface loss induced by subsurface damage, based on the friction generated between micro-interfaces formed by cracks;
- Established a surface loss model that revealed the linear relationship between surface loss and subsurface crack area.

**What are the implications of the main findings?**

- The surface loss mechanism serves as a warning that subsurface damage should be strictly prohibited in the manufacture of high-performance fused silica resonators;
- The surface loss model can offer theoretical guidance and a parameter basis for chemical etching and other fused silica surface treatments.

**Abstract:**

Mitigating the surface loss induced by subsurface damage is of critical importance in the manufacturing of fused silica devices with ultra-low mechanical loss. Chemical etching technology has been applied as an effective approach in controlling surface loss, while fundamental research on its mechanism is still lacking. In this work, chemical etching treatment on lapped fused silica samples was carried out for different periods of time. The evolution of surface micro-topography and roughness was observed. A set of apparatus for the Q-factor measurement was built, and an evaluation method for surface loss was proposed. The changes in the Q-factor and surface loss of samples during the etching process were demonstrated. The mechanism of surface loss induced by subsurface damage was revealed based on the friction of micro-interfaces. A surface loss model was derived from the definition of surface loss and the perspective of kinetic energy conservation. The results show that the surface micro-topography and roughness of samples depend on the evolution of subsurface cracks in etching. The Q-factor of samples is improved by about 50 times via chemical etching, from an initial value of 2.736 × 10^5^ to a maximum value of 1.338 × 10^7^. The subsurface-damage-induced surface loss of the sample lapped in this experiment is about 3.58 × 10^−6^, and most of the loss is caused by cracks in the near-surface layer. This work provides profound insights into the mechanism of surface loss caused by subsurface damage and holds guiding importance for the manufacture of fused silica resonators.

## 1. Introduction

Synthetic high-purity fused silica has been used as a unique structural material for applications demanding precision and stability, such as micro-electro-mechanical systems and solid-wave gyroscopes [1,2,3]. Besides its excellent structural uniformity, low thermal expansion coefficient, and good machinability, the ultra-low intrinsic mechanical loss plays a crucial role in these applications. The intrinsic mechanical loss of fused silica is lower than the 10^−8^ level [4], which enables the mechanical quality factor (Q-factor) of resonators to reach 20 million [5,6]. The Q-factor equals the ratio of the total energy stored in the resonator to the energy loss of the resonator in a vibration cycle, and a higher Q-factor improves the sensitivity and reliability of resonators.

However, this advantage of fused silica in mechanical loss could be overshadowed by three primary energy-dissipation resources in practical application, including air damping, anchor loss, and surface loss [7,8,9]. Air damping is the process in which the moving part loses its kinetic energy due to the resistance or friction provided by the surrounding air. Anchor loss originates from the vibrational energy that leaks out of the resonating structure through its physical supports or anchors into the non-resonating substrate or base. Air damping and anchor loss are both external losses that can be mitigated by maintaining a high vacuum or optimizing the support structure. In contrast, surface loss is an internal loss, which is associated with the surface integrity and state of the resonators. Therefore, for fused silica resonators with optimized structures and operating under high-vacuum conditions, surface loss dominates the loss mechanism.

The mechanism of surface loss is multi-source [10,11,12]. One of the main sources is the subsurface damage induced in the machining stage of resonators. Grinding and lapping are typical methods in the forming process of resonators for their accuracy and efficiency, while the subsurface damage is inevitable because of the material removal mechanism of brittle fracture [13,14]. Experimental results of Penn et al. indicated that even a tiny solitary physical defect on the surface can cause significant surface loss [15]. Lunin et al. took the damaged layer on the glass surface as the deteriorated material whose intrinsic loss was much stronger than that associated with dissipation in the bulk of resonators [16].

As the subsurface damage is a key factor causing surface loss and limiting the Q-factor of resonators, a lot of research on surface treatment was carried out, trying to minimize the influence of subsurface damage on the Q-factor. Heptonstall et al. developed a laser polishing technique that reduced the surface loss of fused silica fibers by 30% [17]. Zeng et al. treated fused silica cylindrical-shell resonators with rounds of chemical etching, and the Q-factor increased dramatically, exceeding 2.5 × 10^7^ [6]. Wang et al. improved the Q-factor of fused silica hemispherical resonators by about 25 times, according to the chemical etching treatment [18]. Ma et al. used a magnetorheological polishing technique to process the surface of fused silica hemispherical resonators, increasing the Q-factor from hundreds of thousands to 2.01 × 10^7^ [19]. Among these surface treatment techniques, chemical etching is most widely used for its effectiveness and efficiency. As a universal surface treatment technology, chemical etching has also shown advantages in improving the optical performance of fused silica [20,21]. However, most of the research on the chemical etching of resonators focuses on optimizing the etching process to pursue a higher Q-factor; the evolution of subsurface damage in etching and its influence on surface loss is less explored. Moreover, the fundamental mechanism of surface loss induced by subsurface damage is of critical importance in designing and manufacturing resonators.

In this work, chemical etching on lapped fused silica samples was carried out to investigate the evolution of subsurface damage and its influence on surface loss. First, the measurement apparatus based on the ring-down method was established for testing the Q-factor. Subsequently, samples were chemically etched for different times, and the surface topography evolution, variation in surface roughness, and the Q-factor were demonstrated. Furthermore, a method of evaluating surface loss was proposed, and the surface loss at different etching stages was calculated. Finally, the mechanism of surface loss induced by subsurface damage was analyzed based on the friction of micro-interfaces, and the evaluation formula of surface loss related to the subsurface crack area was derived.

## 2. Materials and Methods

### 2.1. Sample Preparation

Samples were designed as cylindrical substrates with a diameter of 60 mm and a thickness of 3 mm from 7980-0F fused silica blanks (Corning Incorporated, Corning, NY, USA). The manufacturing sequence comprised four stages: diamond wire slicing, computer numerical control (CNC) contour machining, double-sided lapping, and wet etching. The cylindrical surface of samples was machined on a SPM60 CNC machine (Satisloh AG, Baar, Switzerland) using a metal-bonded diamond grinding wheel with a D15 grain size. To ensure the parallelism of the end faces and dimensional uniformity, all samples were simultaneously subjected to double-sided lapping with loose abrasives. The corresponding lapping process parameters are summarized in Table 1.

Thirty fused silica samples were prepared according to the lapping procedure detailed previously. These samples were divided into ten groups for controlled etching intervals of 0 min, 0.5 min, 1 min, 2 min, 4 min, 7 min, 11 min, 16 min, 22 min, and 29 min.

Chemical etching based on the chemical reaction between hydrofluoric acid (HF) and silica was used to remove the damaged layer, as reported in references [6,18]. The etching solution consisted of a mixture of pure water, HF (20 vol%), and acetic acid (5 vol%). To ensure etching uniformity, the solution temperature was precisely maintained at 26 °C using a temperature-controlled reservoir, and the solution was continuously circulated between the etching tank and the reservoir. Following etching, each sample was thoroughly rinsed with pure water, immersed in pure water for 10 min, and finally dried by hot wind. Sample thickness was measured using a digital thickness gauge with a resolution of 0.001 mm.

### 2.2. Surface Topography and Roughness Characterization

Surface topography and roughness of the samples were characterized using a 3D laser scanning confocal microscope VK-X200 (Keyence Corporation, Osaka, Japan) with a 50× objective. Measurements were performed over a 285 μm × 214 μm field of view. Surface roughness parameters were characterized using areal parameters derived from the full scanned test area. Specifically, Sa (arithmetic mean height) and Sz (maximum height) were used to represent the overall roughness and peak-to-valley variation, respectively. These parameters provide a more comprehensive description of the surface topography compared to profile-based metrics. Sa and Sz for each sample were determined as the arithmetic mean of five measurement results. The final Sa and Sz values were the average of the measurement results of the three samples.

### 2.3. Q-Factor Measurement

The ring-down method serves as an effective means for testing the Q-factor of resonators. The Q-factor was calculated using the following formula [4,22,23]:

(1) Q=πfτ

where *Q* is the value of the Q-factor, and *f* is the natural frequency of the component, and *τ* is the duration from the initial amplitude to its 1/e decay.

**Figure 1:**
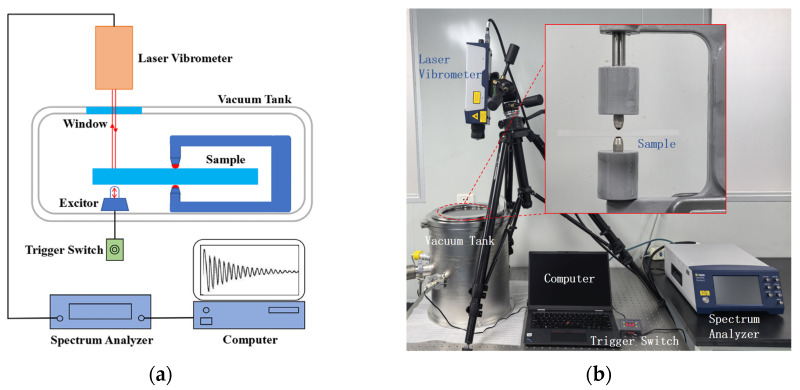
Q-factor measurement system: (**a**) schematic diagram; (**b**) experimental setup.

In our experiments, a Q-factor test system based on the ring-down method was established. The schematic diagram and the experimental setup of this system are shown in Figure 1.

**Figure 2:**
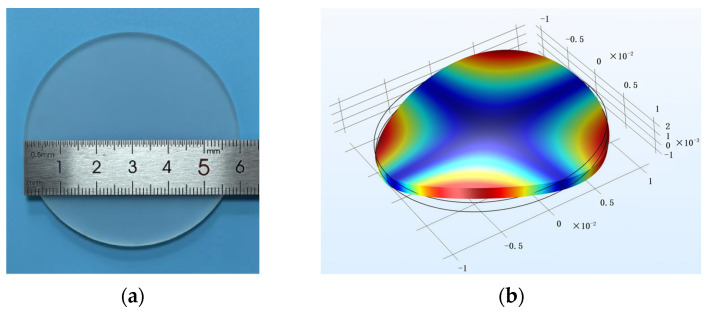
(**a**) Sample picture; (**b**) Modal shape simulation of sample vibrating in the mode of n = 2.

To minimize air damping effects, all tests were carried out in a vacuum tank at an atmospheric pressure below 0.05 Pa. The sample was a fused silica disc (Figure 2a), considering the center of the disc has no displacement along the cylindrical axis when the disc vibrates at its natural frequency. Modal shape simulation (Figure 2b) confirms vibration in its natural vibration mode (n = 2 mode), characterized by four antinodes and four nodal lines. While the circumferential positions of these features may shift depending on the excitation point, the center remains a fixed node.

To locate exactly the center point of the disc, the automatic centering function of the CNC machine was adopted, and the center point was marked with a marker. Two ruby balls with a diameter of 3 mm were used to support the sample. There was a spring device behind the upper ruby, which provided proper pressure on the sample to keep it stable. Vibration of samples was excited by a rigid plastic impactor, which was driven by an electromagnetic trigger. A laser vibrometer VFX-F-110 (Polytec China Ltd., Beijing, China) was applied to collect the vibration information, including the natural frequency, amplitude, and attenuation curve. In the measurements, the Q-factor of each sample was tested three times, and the final Q-factor was taken as the maximum value from the measurement results of the three samples.

## 3. Results

### 3.1. Surface Topography

**Figure 3:**
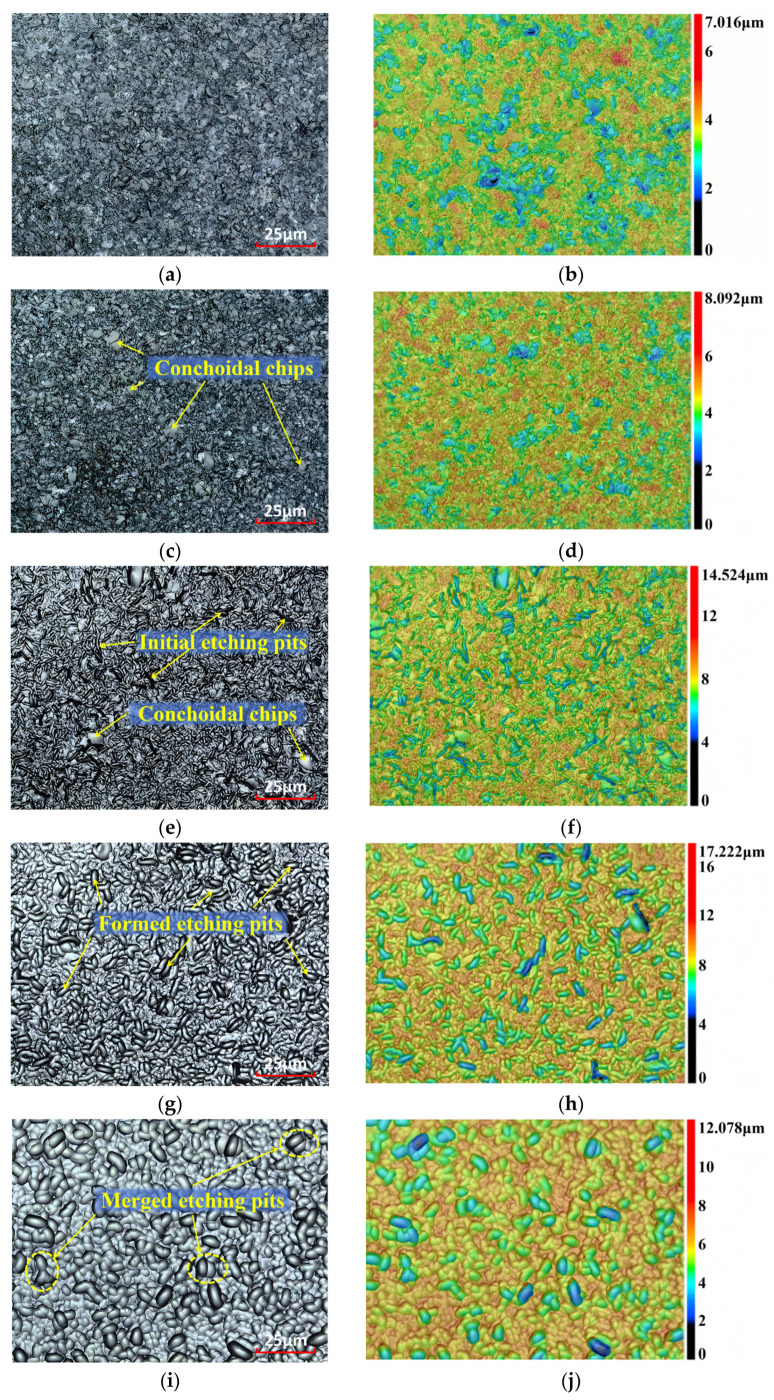
Surface topography evolution with etching time: (**a**) 0 min, 2D; (**b**) 0 min, 3D; (**c**) 0.5 min, 2D; (**d**) 0.5 min, 3D; (**e**) 7 min, 2D; (**f**) 7 min, 3D; (**g**) 16 min, 2D; (**h**) 16 min, 3D; (**i**) 29 min, 2D; (**j**) 29 min, 3D.

The surface topography of the samples after different etching durations is shown in Figure 3, which includes both 2D and 3D images. The 2D images facilitate the observation of textural detail, while the 3D images effectively reflect the local concave and convex areas of the surface topography.

Figure 3a,b show the surface topography of lapped samples prior to etching. Our observations reveal that the lapped surface consists of numerous micro-cracks and conchoidal chips, consistent with the results on loose abrasive lapping of brittle materials based on brittle fracture [24,25]. The micro-cracks and tiny conchoidal chips are intricately intertwined, as shown in Figure 3a, making it difficult to distinguish independent cracks or conchoidal chips. The local concave and convex areas are clearly visible in the corresponding 3D image (Figure 3b).

At the beginning of etching, there is no significant change in surface topography, except that the Sz increases slightly, as seen in Figure 3c,d. With the increase in etching time, the tiny conchoidal chips corroded and dissolved in the etching solution. As a result, cracks that had been hidden under chips were revealed. Meanwhile, the etching solution began to infiltrate into cracks, and the boundaries between cracks and conchoidal chips became more distinct, as shown in Figure 3e,f.

Subsequently, the etching solution penetrated deeper into the cracks. Interfaces formed by cracks were uniformly attacked due to the isotropic nature of fused silica and the non-selective characteristic of chemical wet etching. This process resulted in the enlargement of both crack length and width, forming distinct etching pits. Furthermore, adjacent etching pits expanded in length and width, and eventually coalesced (Figure 3g,h). As the etching process progressed, adjacent pits further merged with each other, as clearly observed in Figure 3i,j. This evolution of surface topography aligns with results reported for the chemical etching of hemispherical resonators and the Ar/CF_4_ plasma etching of fused silica samples [26,27]. These observations indicate that the etched surface topography of fused silica with subsurface damage is predominantly governed by the morphological evolution of cracks during the etching process.

### 3.2. Surface Roughness

**Figure 4:**
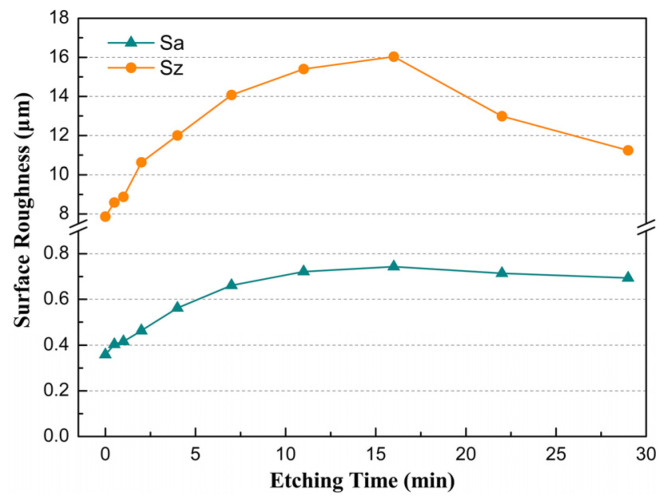
Variations in surface roughness with etching time.

The relationship between the surface roughness of samples and etching time is shown in Figure 4. Both Sz and Sa exhibit similar variations with increasing etching time, although Sz is much greater than Sa. Prior to etching, the lapped surfaces exhibited initial roughness values of Sa = 0.36 μm and Sz = 7.86 μm. In the initial etching stage (0–4 min), both parameters increased linearly with etching time. From 4 min to 16 min, surface roughness continued to rise, but the rate of increase gradually diminished. Maximum values for Sa and Sz, at approximately twice their initial values, were attained at 16 min. Beyond this point, further etching reduced the surface roughness, with Sa and Sz declining to 93% and 70% of their peak values, respectively, by 29 min.

The pre-etched surface roughness of samples was governed by the loose abrasive size, lapping plate hardness, and other process parameters of lapping. The observed temporal evolution of roughness arises from etching-induced topographical changes.

### 3.3. Q-Factor

**Figure 5:**
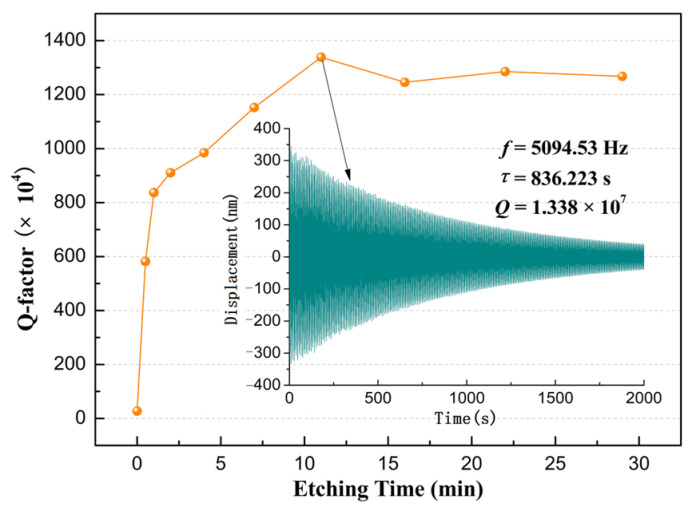
Variation in Q-factor with etching time.

Figure 5 describes the evolution of the sample Q-factor during the etching process. Based on the observed trend, the Q-factor curve can be divided into three regimes: a rapid increase (0~1 min), a gradual increase (1~11 min), and a stable plateau (11~29 min). Notably, the initial Q-factor was 2.736 × 10^5^, rising to 8.367 × 10^6^ after the rapid increase phase, representing an approximately 30-fold enhancement. During the gradual increase stage, the Q-factor exhibited a linear dependence on etching time, reaching its maximum value of 1.338 × 10^7^ at 11 min. After that, the Q-factor stabilized at approximately 1.3 × 10^7^ throughout the plateau stage. The Q-factor was improved by about 48 times throughout the entire etching process.

The observed trend in Q-factor evolution aligns with those reported for fused silica resonators treated by chemical etching or magnetorheological polishing [6,18,19]. This consistency underscores that surface loss constitutes the primary limiting factor for the Q-factor of machined fused silica components. Meanwhile, key differences exist among these experimental results. First, the maximum achieved Q-factors vary: approximately 2.5 × 10^7^ for cylindrical resonators [6], 2 × 10^7^ for hemispherical resonators [18,19], and 1.3 × 10^7^ for the disc samples used in this experiment. This difference may derive from several factors, such as material intrinsic loss, sample geometry, and measurement conditions. A second difference lies in the time required for the Q-factor to reach its maximum from the onset of etching. This is related to the condition of the etching process in which HF concentration and temperature play important roles [28]. The comparison indicates that the etching duration could be shortened by optimizing the etching process parameters to improve the production efficiency of fused silica resonators [6,18,26].

## 4. Discussion

### 4.1. Evolution Mechanism of Subsurface Damage in Etching

**Figure 6:**
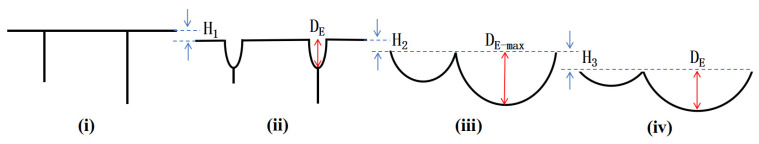
Schematic diagram of the morphological evolution of adjacent cracks in etching: (i) Two adjacent cracks with different depths prior to etching; (ii) Etching pits at initial stage; (iii) Etching pits with maximum depth; (iv) Etching pits at later stage.

The variations in surface topography and roughness in etching indicate that the evolution of subsurface damage plays a leading role. Figure 6 presents a schematic diagram illustrating the morphological evolution of adjacent cracks during etching, elucidating its effect on surface topography and roughness. Figure 6(i) depicts two adjacent cracks with different depths prior to etching. Upon exposure to the etchant (Figure 6(ii)), the etching solution penetrates the cracks, partially opening the interfaces via erosion, and initiating pit formation. Continued etching increases the peak-to-valley roughness (Sz) and pit depth (D_E_) and width, increasing surface roughness, as shown in Figure 4. However, the number of effective cracks contributing to new pit formation decreases with increasing depth [14], resulting in a gradual reduction in the roughness growth rate.

Apparent etching depth (*H_a_*) is defined to characterize the total thickness of the surface layer removed by etching.

(2) $$H_{a}=H_{1}+H_{2}+H_{3}\cdots H_{i}$$

where *H_i_* is the thickness removed by the *i*th etching.

Effective etching depth (*H_e_*) is defined as the maximum depth that the etching solution can reach in a subsurface layer.

(3) $$H_{e}=H_{a}+D_{E}$$

where *D_E_* is the depth of etching pits.

As the lateral etching rate is approximately twice the vertical etching rate, causing the gradual disappearance of the walls between adjacent pits, this process promotes surface planarization. When the competing effects—planarization via pit coalescence and roughening via etching crack enlarging pit depth—achieve a transient balance, etching pits reach the maximum depth (*D_E-max_*) and Sz, as shown in Figure 6(iii) and Figure 4. Further etching leads to a reduction in pit depth (*D_E_*), as shown in Figure 6(iv). This mechanism drives the morphological evolution and the variation in roughness of the silica surface during etching, consistent with the observations for chemically etched fused silica resonators [26,29].

### 4.2. Evaluation of Surface Loss

To further reveal the relationship between surface loss and etching time, surface loss at different etching times is calculated. Surface loss can be calculated from the Q-factor of samples. In the Q-factor measurement, the total loss *Φ* (reciprocal of Q-factor) comprises contributions from multiple sources, including air damping (*Φ_air_*), support loss (*Φ_sup_*), surface loss (*Φ_surf_*), intrinsic loss of material (*Φ_int_*), and other loss (*Φ_oth_*), as expressed by [7]:

(4) $$\Phi =\Phi_{air}+\Phi_{sup}+\Phi_{surf}+\Phi_{int}+\Phi_{oth}$$

In this experiment, only the surface loss decreases during etching, while the other contributions remained constant across measurements. When the Q-factor reached its maximum, the total loss of the sample reached its minimum (*Φ_min_*). Meanwhile, the surface loss, *Φ_surf_*, caused by subsurface damage, substantially reduced the minimum value, *Φ_surf_*_-*min*_:

(5) $$\Phi =\Phi_{min}=\;\Phi_{air}+\Phi_{sup}+\Phi_{int}+\Phi_{oth}+\;\Phi_{surf-min}$$

Then, at any etching time, the total loss of the sample (*Φ_t_*) can be expressed by *Φ_min_* and the surface loss of the sample at the etching time (*Φ_surf,t_*):

(6) $$\Phi_{t}=\Phi_{min}+\Phi_{surf,t}$$

Thus,

(7) $$\Phi_{surf,t}=\Phi_{t}-\Phi_{min}$$

As shown in Figure 6, the Q-factor reaches its maximum value of 1.338 × 10^7^ at 11 min, and the corresponding *Φ_min_* ≈ 0.75 × 10^−7^. Thus, the surface loss at a certain etching time can be calculated as:

(8) $$\Phi_{surf,t}=\Phi_{t}-0.75\times 10^{-7}$$

**Figure 7:**
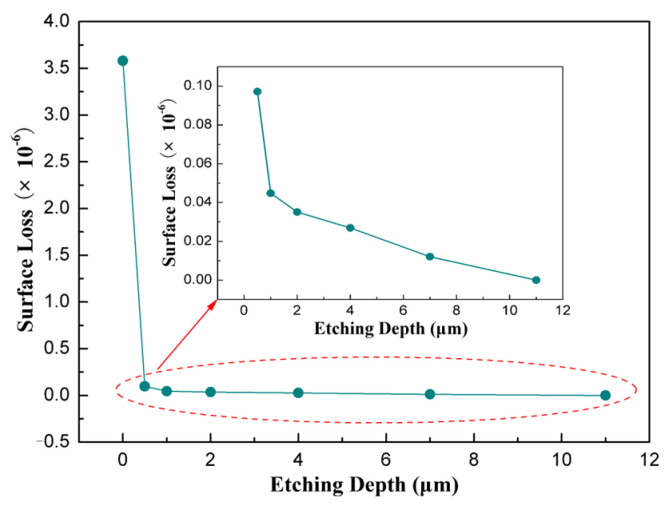
Variation in surface loss with etching depth.

In this experiment, the etching rate of silica can be calculated as 1 μm/min, and the subsurface damage depth of the samples is about 11 μm after etching for 11 min. Taking the etching rate of cracks as a constant, the etching depth at different times can be evaluated, and the surface loss can be calculated according to Equation (8). The relationship between the surface loss and the etching depth is shown in Figure 7.

It can be seen that the surface loss decreases dramatically with the increase in etching depth, especially in the initial stage of etching. After the 0.5 μm surface layer was etched, the surface loss drops from 3.58 × 10^−6^ to 9.72 × 10^−8^, representing a 97% reduction relative to the initial value. In the following etching process, the surface loss reduces slowly, with only an additional 3% reduction in total surface loss.

### 4.3. Surface Loss Mechanism Based on Friction of Micro-Interfaces

The experimental results show that the chemical etching treatment has a significant influence on the surface topography and roughness; meanwhile, the surface loss is mitigated effectively. Based on these findings, this section analyzes the mechanism governing the surface loss.

Firstly, the effect of the surface-to-volume ratio (S/V) on the Q-factor should be discussed. As shown in Figure 3 and Figure 4, sample surfaces become increasingly rugged and rough with etching time up to 16 min. This enhanced roughness increases the effective surface area, thereby elevating the value of S/V. According to reference [30,31], a higher S/V typically reduces the Q-factor. However, the Q-factor of the samples increases and shows no downtrend in the whole etching process. The results indicate that the S/V effect on the Q-factor is not dominant in this experiment.

**Figure 8:**
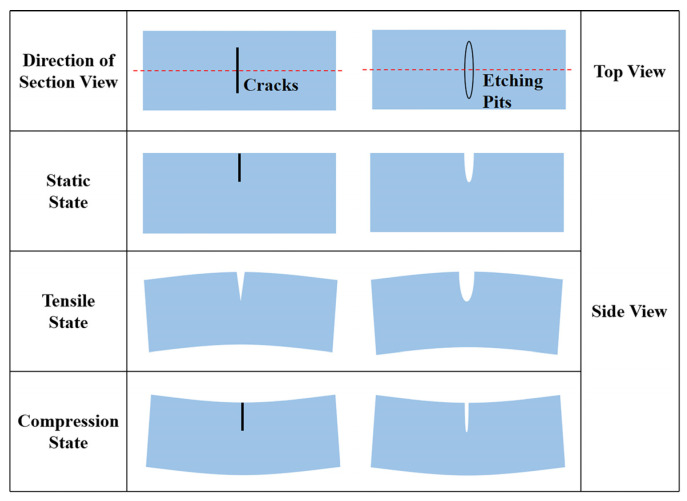
Schematic diagram of deformation of cracks and etching pits under vibration.

Another notable change in the etching process is the evolution from the initial state of a tight-contact micro-interface crack to a state of loose contact, up to the state of open etching pits. Figure 8 illustrates the deformation of cracks and etching pits under vibration. As shown in Figure 8, during vibration, the subsurface zone switches repeatedly between the tensile state and the compression state, where friction occurs when the micro-interfaces in a crack press against each other and cause relative displacement. Each micro-interface becomes a source of energy dissipation, as friction consumes mechanical energy, which is believed to be the dominant mechanism of surface loss induced by subsurface damage. In contrast, open etching pits do not experience this frictional dissipation, as the opposing micro-interfaces are isolated.

### 4.4. A Model of the Relationship Between Surface Loss and Micro-Interfaces

As the friction of micro-interfaces dominates the surface loss induced by subsurface damage, a model is proposed to describe the relationship between the surface loss and micro-interfaces. Based on the definition of surface loss, the formula for the surface loss can be expressed as

(9) $$\Phi_{surf}=\frac{E_{surf}}{2\pi E_{T}}$$

where *E_surf_* is the energy consumed by all micro-interfaces in a single vibration cycle, and *E_T_* is the total energy stored in the resonator. Considering the structural complexity and diversity of resonators, a local mass unit of the vibrating body is taken as the object of analysis. The *E_T_* of the mass unit is equal to its maximum elastic potential energy

(10) $$E_{T}=\frac{1}{2}kA^{2}$$

where *k* is the elastic coefficient, and *A* is the maximum amplitude. To calculate the *E_surf_*, the energy loss *E_i_* per unit area of the micro-interface is first evaluated.

(11) $$E_{i}=\mu Fs$$

where *μ* is the coefficient of friction; *F* is the positive force between interfaces; and *s* is the relative displacement. It is worth noting that *F* and *s* depend on the deformation of interfaces, and this deformation is small, about hundreds of nanometers. As a result, the *F* and *d* are approximately regarded as a linear function of the deformation, thus

(12) F=αΔl s=βΔl

where *α* and *β* are coefficients, and Δ*l* is the deformation. Substituting formula into formula and integrating the consumed energy within the range of maximum deformation, the energy consumed per unit area of the micro-interface in one cycle is

(13) $$E_{i}=4\int_{0}^{A}\mu \alpha l\beta dl=2\mu \alpha \beta A^{2}$$

Assuming that the total area of the micro-interfaces contained in the mass unit is *S*, the total energy dissipation of the mass unit is

(14) $$E_{surf}=SE_{i}=2S\mu \alpha \beta A^{2}$$

Thus,

(15) $$\Phi_{surf}=\frac{2S\mu \alpha \beta A^{2}}{2\pi \frac{1}{2}kA^{2}}=\frac{2S\mu \alpha \beta }{\pi k}$$

It should be noted that the linear assumptions in Equations (11) and (12) represent a simplification of the complex contact behavior at micro-interfaces. These assumptions are based on the small-deformation regime characteristic of high-Q resonators, where the deformation at the micro-interface is on the order of hundreds of nanometers, and the mass unit can be treated as an ideal elastic body following Hooke’s law. Under such conditions, a linear approximation is physically reasonable as a first-order description. Furthermore, the derived model predicts that surface loss is independent of vibration amplitude, which is consistent with our experimental observation that the measured Q-factor remains stable across different initial excitation amplitudes. This consistency provides indirect support for the linear assumptions. Formula (15) shows that the surface loss has a linear relationship with the total area of the micro-interfaces. Our previous work showed that the area of micro-interfaces decreased as an exponential function of depth [14]. It is indicated that the surface loss also has an exponential relationship with the subsurface damage depth, which coincides well with the results shown in Figure 7.

While quantitative metrics, such as pit area fraction, pit density, and depth distribution, would provide a more detailed characterization of surface topography evolution, the high density of cracks and etching pits—combined with the continuous nature of the chemical etching process—make an accurate extraction of these parameters challenging. In particular, the merging of adjacent cracks during etching complicates the determination of individual crack dimensions and counts. Therefore, the present study focuses on the evolution of surface morphology and roughness as effective indicators of subsurface crack behavior, which, together with Q-factor measurements, elucidate the mechanism of surface loss mitigation. Establishing a direct quantitative relationship between Q-factor or surface loss and crack/pit metrics remains a key objective for future work, for which techniques such as magnetorheological polishing or chemical mechanical polishing may offer greater suitability.

## 5. Conclusions

In this work, chemical etching treatment on lapped fused silica samples was carried out to investigate the evolution of subsurface damage and its influence on surface loss. An evaluation method for surface loss based on the Q-factor measuring apparatus was proposed. The experimental results show that the morphological evolution of subsurface cracks dominates the surface topography, surface roughness, Q-factor, and surface loss of samples in the whole etching process. The surface of the samples becomes uneven and is eventually covered by elliptical etching pits. Surface roughness initially increases, and reaches its maximum—approximately double the initial values—then shows a downward trend. The Q-factor exhibits remarkable growth after etching treatment, increasing from 2.736 × 10^5^ to 1.338 × 10^7^. The variation in surface loss with etching depth indicates that 97% of the total surface loss is caused by cracks in the near-surface layer and that in the deeper layer contributes to the remaining 3%. Friction of micro-interfaces formed by subsurface cracks is the dominating mechanism of surface loss. The surface loss evaluation model elucidates the linear relationship between surface loss and subsurface crack area. This work could offer theoretical guidance for the chemical etching process and for high-performance manufacturing of fused silica resonators.

## Figures and Tables

**Table 1:** Process parameters of lapping.

Process Parameter	Value
Lapping pressure (kg/cm^2^)	0.06
Diameter of plate (mm)	640
Plate rotation speed (r/min)	10
Mesh number of alumina abrasives	1000
Flow rate of slurry (L/min)	8

## Data Availability

The data that support the findings of this study are available from the corresponding author upon reasonable request.

## References

[1] Nagourney T., Cho J.Y., Shiari B., Darvishian A., Najafi K. 259 Second Ringdown Time and 4.45 million Quality Factor in 5.5 kHz Fused Silica Birdbath Shell Resonator. Proceedings of the IEEE Transducers.

[2] Maslov A.A., Maslov D.A., Ninalalov I.G., Merkuryev I.V. (2023). Hemispherical Resonator Gyros (An Overview of Publications). Gyroscopy Navig..

[3] Rozelle D.M. (2009). The Hemispherical Resonator Gyro: From Wineglass to the Planets. Adv. Astronaut. Sci..

[4] Ageev A., Palmer B.C., Felice A.D., Penn S.D., Saulson P.R. (2004). Very High Quality Factor Measured in Annealed Fused Silica. Class. Quantum Gravity.

[5] Numata K., Yamamoto K., Ishimoto H., Otsuka S., Kawabe K., Ando M., Tsubono K. (2004). Systematic Measurement of the Intrinsic Losses in Various Kinds of Bulk Fused Silica. Phys. Lett. A.

[6] Zeng L., Pan Y., Luo Y., Xiao P., Liu J., Tan Z., Yang K., Luo H. (2021). Fused Silica Cylindrical Shell Resonators with 25 million Q Factors. J. Phys. D Appl. Phys..

[7] Xi X., Wu X., Wu Y., Zhang Y. (2017). Modeling and Analysis of Mechanical Quality Factor of the Resonator for Cylinder Vibratory Gyroscope. Chin. J. Mech. Eng..

[8] Pan Y., Su P., Zhang Q., Xiao Y., Zhang H., Li M. (2025). Research Progress on Energy Dissipation Mechanisms in MEMS Resonators: A Review. IEEE Sens. J..

[9] Wang A., Sahandabadi S., Harrison T., Spicer D., Ahamed M.J. (2022). Modelling of Air Damping Effect on the Performance of Encapsulated MEMS Resonators. Microsyst. Technol..

[10] Lunin B.S., Tokmakov K.V. (2022). The Formation of Microcrystalline Defects on the Surface of Silica Glass Arising from Mechanochemical Processing. Int. J. Appl. Glass Sci..

[11] Lunin B.S., Torbin S.N. (2001). On the Mechanism of Acoustic Losses in the Hydroxylated Surface Layer of Silica Glass. Moscow Univ. Chem. Bull..

[12] Bilenko I.A., Braginsky V.B., Lourie S.L. (2004). Mechanical Losses in Thin Fused Silica Fibres. Class. Quantum Gravity.

[13] Gao S., Li H., Huang H., Kang R. (2022). Grinding and Lapping Induced Surface Integrity of Silicon Wafers and Its Effect on Chemical Mechanical Polishing. Appl. Surf. Sci..

[14] Zhang X.Q., Song X.F., Sun Y.C., Du X.R., Zhang C.Y., Zu C.K. Distribution Characteristics of Subsurface Damage Induced by Different Machining Methods of Fused Silica. Proceedings of the SPIE 11568: Applied Optics and Photonics, Optics Ultra Precision Manufacturing and Testing.

[15] Penn S.D., Harry G.M., Gretarsson A.M., Kittelberger S.E., Saulson P.R., Schiller J.J., Smith J.R., Swords S.O. (2001). High Quality Factor Measured in Fused Silica. Rev. Sci. Instrum..

[16] Lunin B.S., Lopatin V.M. (2022). Surface Internal Friction in High-Q Fused Quartz Resonators. Inorg. Mater..

[17] Heptonstall A., Barton M.A., Bell A.S., Bohn A., Cagnoli G., Cumming A., Grant A., Gustafson E., Hammond G.D., Hough J. (2014). Enhanced Characteristics of Fused Silica Fibers Using Laser Polishing. Class. Quantum Gravity.

[18] Wang C., Ning Y., Zhao W., Yi G., Huo Y. (2023). Surface Evolution of Fused Silica Hemispherical Resonators and Its Influence on the Quality Factor. Sens. Actuators A Phys..

[19] Ma C., Liu H., Chen M., Cheng J., Tian J., Qin B., Sun J., Zhou Z., Guo J. (2025). Effect of Surface Integrity on Quality Factor of Hemispherical Resonator. Int. J. Mech. Sci..

[20] Kamimura T., Akamatsu S., Horibe H., Shiba H., Motokoshi S., Sakamoto T., Jitsuno T., Okamato T., Yoshida K. (2004). Enhancement of Surface-Damage Resistance by Removing Subsurface Damage in Fused Silica and Its Dependence on Wavelength. Jpn. J. Appl. Phys..

[21] Liu H., Ye X., Zhou X., Huang J., Wang F., Zhou X., Wu W., Jiang X., Sui Z., Zheng W. (2014). Subsurface Defects Characterization and Laser Damage Performance of Fused Silica Optics during HF-etched Process. Opt. Mater..

[22] Numata K., Bianc G.B., Ohishi N., Sekiya A., Otsuka S., Kawabe K., Ando M., Tsubono K. (2000). Measurement of the Intrinsic Mechanical Loss of Low-Loss Samples Using a Nodal Support. Phys. Lett. A.

[23] Nasyrov R.S., Lunin B.S., Lopatin V.M. (2017). Preparation of Silica Glass with a Low Degree of Internal Friction. Glass Phys. Chem..

[24] Neauport J., Destribats J., Maunier C., Ambard C., Cormont P., Pintault B., Rondeau O. (2010). Loose Abrasive Slurries for Optical Glass Lapping. Appl. Opt..

[25] Lv X., Yuan J.L., Wen D.H. (2009). Comparison of Processing Features between Semi Bonded Abrasive Lapping and Loose Abrasive Lapping. Key Eng. Mater..

[26] Chu J., Liu X., Liu C., Zhang J., Xiao J., Wang X., Chen X., Xu J. (2022). Fundamental Investigation of Subsurface Damage on the Quality Factor of Hemispherical Fused Silica Shell Resonator. Sens. Actuators A Phys..

[27] Jin H., Xin Q., Li N., Jin J., Wang B., Yao Y. (2013). The Morphology and Chemistry Evolution of Fused Silica Surface after Ar/CF_4_ Atmospheric Pressure Plasma Processing. Appl. Surf. Sci..

[28] Shu Y., Duan W., Jiao C. (2019). SSD Evolution Model in HF Etching of Fused Silica Optics. OPTIK.

[29] Zeng L., Tao Y., Pan Y., Liu J., Yang K., Luo H. (2021). Experimental Study on Variation of Surface Roughness and Q Factors of Fused Silica Cylindrical Resonators with Different Grinding Speeds. Micromachines.

[30] Startin W.J., Beilby M.A., Saulson P.R. (1998). Mechanical Quality Factors of Fused Silica Resonators. Rev. Sci. Instrum..

[31] Gretarsson A.M., Harry G.M. (1999). Dissipation of Mechanical Energy in Fused Silica Fibers. Rev. Sci. Instrum..

